# Stochastic Spatio-Temporal Dynamic Model for Gene/Protein Interaction Network in Early *Drosophila* Development

**Published:** 2009-10-19

**Authors:** Cheng-Wei Li, Bor-Sen Chen

**Affiliations:** Laboratory of Systems Biology, National Tsing Hua University, Hsinchu, 300, Taiwan. Email: bschen@ee.nthu.edu.tw

**Keywords:** 3-D embryo space-time dynamic model, gene/protein interaction network, *drosophila* embryo, eve stripe formation, transcription regulation, diffusion mechanism

## Abstract

In order to investigate the possible mechanisms for eve stripe formation of *Drosophila* embryo, a spatio-temporal gene/protein interaction network model is proposed to mimic dynamic behaviors of protein synthesis, protein decay, mRNA decay, protein diffusion, transcription regulations and autoregulation to analyze the interplay of genes and proteins at different compartments in early embryogenesis. In this study, we use the maximum likelihood (ML) method to identify the stochastic 3-D Embryo Space-Time (3-DEST) dynamic model for gene/protein interaction network via 3-D mRNA and protein expression data and then use the Akaike Information Criterion (AIC) to prune the gene/protein interaction network. The identified gene/protein interaction network allows us not only to analyze the dynamic interplay of genes and proteins on the border of eve stripes but also to infer that eve stripes are established and maintained by network motifs built by the cooperation between transcription regulations and diffusion mechanisms in early embryogenesis. Literature reference with the wet experiments of gene mutations provides a clue for validating the identified network. The proposed spatio-temporal dynamic model can be extended to gene/protein network construction of different biological phenotypes, which depend on compartments, e.g. postnatal stem/progenitor cell differentiation.

## Introduction

An early embryonic stage in *Drosophila* embryogenesis, i.e. the syncytial blastoderm stage, is completed two hours after the onset of fertilization and periodic segments are then characterized. Before the determination of periodic segments, the embryo is not yet separated by membranes, and macromolecules such as transcription factors (TFs) can diffuse freely and regulate downstream target genes in neighboring nucleus. Hence, at the syncytial blastoderm stage diffusion mechanism is fast enough to vary the concentrations of TFs in transcription regulations. Through a series of high/low affinity bindings of transcription regulations, downstream genes are dictated to express in their corresponding space of an embryo. Therefore, we assume that the transcription regulation and diffusion mechanism may play a cooperative role in characterizing embryonic segments.

Although some topics about protein diffusion have been well studied,^1^,^2^ gradient dynamics of concentrations of transcription factors is still hard to be analyzed without quantitative inference under dynamic modeling. For example, critical boundaries settled by protein concentration gradient in dynamic models of early embryogenesis have allowed investigators to re-examine quantitatively concentration gradient dynamics.^3^ Jaeger and his colleagues have used mRNA spatial-temporal data and dynamic model to characterize the establishment of gap domains.^4^ Therefore, in order to analyze the diffusion mechanisms of transcription factors at different domains of *Drosophila* embryo, a spatio-temporal model is needed. In recent studies, early embryogenesis in *Drosophila* includes at least 31 genes in subdividing the embryonic patterns into 14 segmental primordia along the anterior-posterior (A-P) axis .^5^ In the past several decades, the spatio-temporal expressions of the early development-related genes (*bicoid* (*bcd*), *caudal* (*cad*), *hunchback* (*hb*), *giant* (*gt*), *knirps* (*kni*), *Krüppel* (*Kr*), *tailless* (*tll*), *even-skipped* (*eve*), *fushitarazu* (*ftz*), *hairy*, *odd-skipped* (*odd*), *paired* (*prd*), *runt* and *sloppy-paired* (*slp*)) have been provided and studied during the early developmental stages of *Drosophila melanogaster*. The 14 early development-related genes can be roughly divided into three classes, i.e. maternal genes, gap genes and pair-rule genes, which have been regarded as hierarchical transcription regulations with positive auto-regulations to generate and refine the constitutions of segments.^6^,^7^ At the beginning of early embryogenesis, gap genes are regulated by high-level expressions of maternal TFs to initiate an early embryo development. Gene expression boundaries are determined by thresholds of protein concentration, while gene expression borders are refined by autoregulation and repression.^8^,^9^

Three classified genes (i.e. maternal genes, gap genes and pair-rule genes) into which the 14 early development-related genes can be divided are described in detail in the following. The maternal genes, i.e. *bcd*, *cad* and *hb*, diffuse and regulate gap genes with different expression levels in each spatial region along the A-P axis of the *Drosophila* embryo. The gap genes, i.e. *gt*, *hb*, *kni*, *Kr* and *tll*, define roughly the differences between two neighboring stripes by protein diffusion. The pair-rule genes, i.e. *eve*, *ftz*, *hairy*, *odd*, *prd*, *runt* and *slp*, define periodic patterns of the embryo by transcription regulation and protein diffusion. Two of these pair-rule genes, i.e. *eve* and *ftz*, are involved in defining even and odd segments of the 14 segmental primordia along the A-P axis.^10^,^11^ The odd and even segments of concern are the seven eve stripes and seven ftz stripes, respectively. Moreover, at the blastoderm stage, along the D-V axis, three main regions, i.e. non-neural ectoderm (prospective epidermis), neurectoderm (prospective nervous system and larval ventral epidermis) and mesoderm (prospective muscle and connective tissue) are also divided.^12^ The genes, which determine the three primary regions along the D-V axis, are different from these 14 early development-related genes which determine periodic segments along the A-P axis. In this study, for the convenience of analysis and system identification we will define spatial regions in the two-dimensional (2-D) space of the embryo along the A-P and D-V axes according to the above information. However, we only analyze the A-P formation of embryo after system modeling of transcriptional regulatory network, and the D-V formation can be analyzed by a similar procedure.

At the early developmental stages of *Drosophila*, the three-dimensional (3-D) spatio-temporal expression data of 14 early development-related proteins (http://flyex.ams.sunysb.edu/flyex/),^13^–^16^ genome-wide mRNA time-course expression data^17^ and mRNA 3-D spatio-temporal expression data (http://flyex.ams.sunysb.edu/lab/gaps.html)^4^,^6^ have been published and can be used for a system dynamic modeling of early *Drosophila* development. Interestingly, by comparing the normalized protein spatio-temporal expression data with mRNA spatio-temporal expression data, the trends of gene expressions along the A-P axis are found.^3^ In this study, we incorporate the mRNA 3-D data with protein 3-D data to construct the gene/protein interaction network for the transcription regulations and diffusion mechanisms of early embryogenesis via our stochastic 3-D dynamic model. However, there are some expression data within the 14 early development-related genes are unavailable in mRNA 3-D spatio-temporal expression database. In recent studies, Neural-Network (NN) model, which could be trained to optimize its internal network to learn the behaviors of complex systems, has been used to not only infer gene network regulatory relationships based on genome-wide microarray data^18^ but also build the relationship between input and output information by using a back-propagation algorithm to learn from the training data.^19^–^22^ Therefore, for the unavailable mRNA data, we will use the back-propagation NN training method to obtain the mimic mRNA data according to the available protein and mRNA 3-D data.

In recent years, since the development of experimental techniques has increased the quality and amount of available mRNA and protein expressions, many approaches, e.g. fuzzy logic,^23^,^24^ recurrent neural networks,^25^–^27^ Bayesian networks,^28^,^29^ Boolean networks^30^,^31^ and differential equations,^32^–^34^ have been widely exploited to unravel regulation networks from the perspective of systems biological. For the well available protein spatio-temporal data in early *Drosophila* development, nonlinear 2-D dynamic models have been employed to analyze the transcription regulation properties and effect of gap genes on eve stripe formation.^3^,^6^,^16^,^35^–^38^ However, more efforts are needed to incorporate these pathways and gene networks with a spatio-temporal gene/protein interaction network to interpret the dynamic system behavior in early *Drosophila* development since not only protein but also mRNA 3-D spatio-temporal data are both available for dynamic interplay of genes and proteins at different compartments of *Drosophila* embryo in early embryogenesis. The mechanisms of early *Drosophila* development in the whole embryo can be unraveled clearly if the dynamic interactions of genes and proteins are considered at different compartments in early embryogenesis. Therefore, in this study, we propose a stochastic 3-D dynamic model for constructing the gene/protein interaction network of early *Drosophila* development.

In this study, we focus on the topic of investigating the possible mechanisms for the eve stripe formation of *Drosophila* embryo. In this biological development approach, it is assumed that transcription regulations consist of cis-effect and trans-effect. Since edges, i.e. transcription regulations, in a gene regulatory network must be constantly selected in order to survive randomization forces, trans-effects, which are the binding affinities of specific transcription factors to cis-regulatory regions in the promoter of the target gene, would be varied rapidly while cis-effects, which are regulated directly by physical attachment of TF’s binding cis-regulatory regions, are relatively fixed.^39^ Thus, we assume that regulation abilities, i.e. trans-effects, should vary with different spatial regions of the embryo, which results from different binding affinities of diffusible TFs. Based on the constructed stochastic 3-D Embryo Space-Time model (stochastic 3-DEST model), we analyze the transcription regulations and diffusion mechanisms for gene/protein interaction network. The stochastic 3-DEST model with 28 state variables is employed to represent the transcription/translation regulation process between 14 mRNA genes and the corresponding TFs in early embryogenesis. Moreover, because we consider both the environmental noises and the intrinsic noises in mRNA and protein data, stochastic partial differential equations (PDEs) are employed for the transcriptional and translational regulatory model of early embryogenesis. In order to understand the roles of TFs in each spatial region, according to the signs of diffusion parameters of the stochastic 3-DEST model, a TF can be considered as a donor (>0) or an acceptor (<0) in each spatial region to balance instant concentrations of the whole embryo. Hence, the TF in a spatial region that diffuses to (from) the neighboring spatial regions, is called a donor (acceptor). In addition, from previous studies we know that transcription regulations can be inferred by a dynamic model via microarray data.^33^,^36^,^40^ However, how to sieve out the insignificant transcription regulations from the whole gene/protein interaction network is still a problem. For this reason, according to the stochastic 3-DEST model, the Akaike Information Criterion (AIC)^41^ for model order detection combined with the maximum likelihood (ML) for parameter estimation in system identification is used in this study to detect significant upstream regulators and to prune insignificant transcription regulations for refining the gene/protein interaction network of early *Drosophila* development. From the identified stochastic 3-DEST model, we can not only find the significant transcription regulations of the corresponding TFs, which control the anterior/posterior border formation of eve stripes, but also validate these results with wet experiments. In order to validate the identified effect of transcription regulation and diffusion on early *Drosophila* development, the wet experiments, i.e. gene mutations,^7^,^9^,^10^,^42^–^46^ regulatory module classification^47^ and cis-regulatory module detection,^48^ have been employed to trace back the direct or indirect transcription regulations and protein diffusions in early *Drosophila* development. From the perspective of the network motifs of the identified gene/protein interaction network in the embryo, we find that transcription regulations and protein diffusion mechanisms may play a cooperative role in the formation of eve stripes in early *Drosophila* development.

## Methods

### System modeling and identification for gene/protein interaction network

To identify the dynamic behavior of the early development-related genes, the procedure of system identification in early embryogenesis is divided into four steps. First, utilizing fully the well-published spatio-temporal data and the prior knowledge of early embryogenesis, we construct a stochastic 3-DEST model to identify the molecular dynamics of gene/protein interaction network in early embryogenesis. Second, for system modeling, we use Eve’s spatial expression at the cleavage cycle 14A temporal class 8 (c14A8) of the nuclear cleavage to settle stripe boundaries and region boundaries of each stripe for dividing the embryo into seven eve stripes along the A-P axis and into three spatial regions (i.e. anterior part, middle part and posterior part) along the D-V axis, respectively. Third, for the early development-related genes, since a part of the mRNA spatio-temporal data are unavailable, we incorporate the available mRNA and protein spatio-temporal expression data with the back-propagating NN training method to train and simulate the mimic data for the unavailable mRNA spatio-temporal expression data (see Appendix I). Fourth, we identify the model parameters and select the significant regulatory parameters for the stochastic 3-DEST model to construct the transcriptional regulatory network in every spatial region by the ML estimation method and the AIC backward elimination method, respectively. Finally, the transcriptional regulatory networks in every spatial region are connected together to construct the entire spatio-temporal gene/protein interaction network for early *Drosophila* development.

**Remark:** If the information of cooperation bindings is richer in future, the transcriptional regulations due to cooperation binding can be easily extended to the regulation candidates of the 3-DEST model, which can improve the proposed model of gene/protein network but with increased computation burden when using the AIC method in early embryogenesis.

### Stochastic PDEs model in eve stripe formation

In previous studies, dynamic models with protein synthesis, protein diffusion and protein decay have been utilized in the description of the mechanism of embryonic development.^3^,^4^,^6^,^35^–^38^ To analyze the dynamic interplay of genes and proteins in early embryogenesis, six stochastic molecular dynamics are incorporated in the 3-DEST model, i.e. (1) protein synthesis, (2) protein decay, (3) mRNA decay, (4) protein diffusion, (5) transcription regulations, and (6) autoregulation. In addition, in order to differentiate mRNA expressions from protein expressions, we define two state variables *X_i_* and *Y_i_* to represent the 3-D spatio-temporal mRNA profiles of the *i*th target gene and its corresponding TFs, respectively. According to the transcription regulation model proposed in previous studies,^6^,^33^,^36^,^40^ the stochastic 3-DEST model for the *i*th target gene and their upstream regulatory TFs in the gene/protein interaction network of *Drosophila* development is proposed as follows:

(1) $$\begin{array}{l} \frac{\partial X_{i}(t,x,y)}{\partial t}=\kappa_{i}(x,y)-\alpha_{i}(x,y)X_{i}(t,x,y)\\ \;\;\;\;\;\;\;\;\;\;\;\;\;\;\;\;\;\;\;\;\;\;\;\;\;+\sum_{j=1}^{14}\beta_{ij}(x,y)f(Y_{j}(t-\tau_{j},x,y))\\ \;\;\;\;\;\;\;\;\;\;\;\;\;\;\;\;\;\;\;\;\;\;\;\;\;+\upsilon_{i}(t,x,y)\\ \frac{\partial Y_{j}(t,x,y)}{\partial t}=\varpi_{j}(x,y)+\alpha_{j}(x,y)X_{i}(t,x,y)\\ \;\;\;\;\;\;\;\;\;\;\;\;\;\;\;\;\;\;\;\;\;\;\;\;-\lambda_{j}(x,y)Y_{j}(t,x,y)\\ \;\;\;\;\;\;\;\;\;\;\;\;\;\;\;\;\;\;\;\;\;\;\;\;+\gamma_{j}(x,y)\nabla^{2}Y_{j}(t,x,y)+\zeta_{j}(t,x,y),\\ \;\;\;\;\;\;\;\;\;\;\;\;\;\;\;\;\;\;\;\;\;\;\;\;\;i,j=1,2,\ldots ,14 \end{array}$$

where *X_i_*(*t*, *x*, *y*) represents the mRNA expression of the *i*th target gene, *Y_j_*(*t*, *x*, *y*) denotes the expression of the *j*th TF of the target gene, and *f(Y_j._*(*t*, *x*, *y*)) defined as *f*(*Y*) = *Y^n^*/(*P^n^* *+ Y^n^*) is a sigmoid function to denote the regulatory bindings of TFs on the promoters of targets.^39^,^49^,^50^ Here, *P* is defined as the means of protein expressions, which imply cis-effects of transcription regulations. The term $\Sigma_{j=1}^{14}\beta_{ij}(x,y)f(Y_{j}(t-\tau_{j},x,y))$ denotes the transcription regulation, i.e. trans-effect, on the *i*th target gene from its TFs. *α_i_*(*x*, *y*) stands for mRNA decay rate for the *i*th gene and is equal to the synthesis rate of the *i*th protein, and *λ_j_*(*x*, *y*) stands for protein decay rate. *κ_i_*(*x*, *y*) and *ϖ_j_*(*x*, *y*) are basal level of mRNA and protein generation, respectively, and they satisfy *κ_i_*(*x*, *y*), *ϖ_j_*(*x*, *y*) ≥ 0. The diffusion operator ▿^2^ = *∂*^2^/*∂x*^2^ + *∂*^2^/*∂y*^2^ is the Laplacian operator in 2-D to denote the diffusion of protein at the location (*x*, *y*). In Eq. (1), mRNA expressions are transcriptionally regulated by TFs (i.e. $\Sigma_{j=1}^{14}\beta_{ij}(x,y)f(Y_{j}(t-\tau_{j},x,y)$ and translated for protein synthesis *α_i_*(*x*, *y*) *X_i_*(*t*, *x*, *y*) in the downstream translation process. In the second equation of Eq. (1), the *j*th TF, *Y_j_*(*t*, *x*, *y*), is assumed to be produced in the translation process by the corresponding mRNA *α_i_*(*x*, *y*) *X_i_*(*t*, *x*, *y*) from the upstream transcription process and decayed by degradation *λ_j_*(*x*, *y*) *Y_j_*(*t*, *x*, *y*) and diffusion *γ_j_*(*x*, *y*)▿^2^*Y_j_*(*t*, *x*, *y*).^51^ Diffusion coefficients of the *j*th TF are represented by *γ_j_*(*x*, *y*). *β_ij_*(*x*, *y*) denotes the regulatory ability of the *j*th TF (or regulatory protein), *Y_j_*, on the promoter region of the target gene *X_i_*. *β_ij_*(*x*, *y*) > 0 stands for the *i*th target gene activated by the *j*th TF (prospective activator) or not repressed by the *j*th TF (prospective repressor) while *β_ij_*(*x*, *y*) < 0 stands for the *i*th target gene not activated by the *j*th TF (prospective activator) or repressed by the *j*th TF (prospective repressor).^39^ Therefore, the gene/protein interaction network of early *Drosophila* development is constructed by linking up all target genes through the regulations of their upstream TFs, $\Sigma_{j=1}^{14}\beta_{ij}(x,y)f(Y_{j}(t-\tau_{j},x,y))$ in Eq.(1). Moreover, the productions of *Y_j_* in Eq.(1)are synthesized by the corresponding mRNA *X_j_* and diffused from *Y_j_* in the neighborhood. Model uncertainty, fluctuations of the basal levels and measurement noises in the mRNA (transcription) dynamics and protein (translation) dynamics are denoted by stochastic noise *υ_i_*(*t*, *x*, *y*) and *ζ_j_*(*t*, *x*, *y*), respectively. *x* and *y* denote the location of the embryo in the 2-D space, i.e. the coordination in the x-axis and y-axis.

**Remark:** The dynamic model in Eq. (1) is to interpret the transcription/translation regulation processes of 14 genes in early embryogenesis. The first Equation of Eq. (1) describes the transcription regulation of the *i*th gene; and the mRNA productive rate is mainly due to the transcription regulations of 14 proteins (i.e. TFs), the influence of basal level and degradation of mRNA. The noise *υ_i_*(*t*, *x*, *y*) denotes the fluctuation of basal level, measurement noise and modeling residue. Since the expression levels of TFs can be altered with different spatial regions of the whole embryo by diffusion mechanism, the relationship of transcription regulation between one TF and its target gene is also different in different spatial regions. The second equation of Eq. (1) describes protein production in the translational diffusion process at the location (*x*, *y*). The protein productive rate is mainly influenced by the translation of mRNA, diffusion from the neighboring space, and degradation rate of the protein. The noise *ζ_j_*(*t*, *x*, *y*) is due to the fluctuation of the basal level of protein, measurement noise and modeling error. The model in Eq. (1) describes the interplay of gene/protein interactions at the location (*x*, *y*). The parameters of the stochastic spatio-temporal dynamic model in Eq. (1) can be estimated by the spatio-temporal profile of mRNA data and protein data in each spatial region. The regulatory gene/protein network can be linked gene by gene through the transcription regulations $\Sigma_{j=1}^{14}\beta_{ij}(x,y)f(Y_{j}(t-\tau_{j},x,y))$ to other regulatory TFs iteratively.

In the proposed stochastic dynamic model in Eq. (1), the interplay of six stochastic processes, i.e. protein synthesis, protein decay, mRNA decay, protein diffusion, transcription regulations and autoregulation, mimics the dynamics in early embryogenesis. We are the first to combine mRNA dynamic equations with protein dynamic equations to mimic the dynamic interaction network of target genes and their regulatory proteins via 3-D mRNA and protein data at different compartments in early *Drosophila* development. Our main purpose is to infer the possible mechanisms of eve stripe formation by investigating the estimated parameters *κ_i_*, *ϖ_j_*, *α_j_*, *β_ij_*, *λ_j_* and *γ_j_*, *i* = 1, 2, ..., 14 of the system dynamic model in Eq. (1) via mRNA and protein data. Since it is hard to solve directly the identification problem of the continuous 3-DEST model in Eq. (1), we discretize the continuous 3-DEST model in Eq. (1)^52^ and the location (*x*, *y*) on the continuous plane is transformed into the location (*l*, *m*) on the discrete plane. The discrete 3-DEST model is shown as follows:

(2) $$\begin{array}{l} X_{i}(k+1,l,m)=d_{i,l,m}+(1-a_{i,l,m})X_{i}(k,l,m)+\sum_{j=1}^{14}b_{ij,l,m}f(Y_{j}(k-k^{\prime },l,m))+{ɛ}_{i}(k,l,m)\\ Y_{i}(k+1,l,m)=w_{i,l,m}+a_{i,l,m}X_{j}(k,l,m)+c_{j,l,m}Y_{j}(k,l,m)\;\;\;\;i,j=1,2,\ldots ,14\\ \;\;\;\;\;\;\;\;\;\;\;\;\;\;\;\;\;\;\;\;\;\;\;\;\;\;\;\;+\rho_{j,l,m}(\frac{Y_{j}(k,l,-1,m)-2Y_{j}(k,l,m)+Y_{j}(k,l+1,m)}{h_{x}^{2}}\;\;\;\;l=1,2,3\\ \;\;\;\;\;\;\;\;\;\;\;\;\;\;\;\;\;\;\;\;\;\;\;\;\;\;\;\;\;+\frac{Y_{j}(k,l,m-1)-2Y_{j}(k,l,m)+Y_{j}(k,l,m+1)}{h_{y}^{2}})+\delta_{j}(k,l,m)\;\;\;\;m=1,2,\ldots ,21 \end{array}$$

with the stability constraints *d_i_*_,_*_l_*_,_*_m_* ≥ 0, *w_j_*_,_*_l_*_,_*_m_* ≥ 0, $\left|1-a_{j,l,m}\right|\leq 1,\{\begin{array}{cc} |c_{j,l,m}-4\rho_{j,l,m}(h_{x}^{-2}+h_{y}^{-2})|\leq 1 & \text{if}\;\rho_{j,l,m}<0\\ \left|c_{i,l,m}\right|\leq 1 & \text{if}\;\rho_{j,l,m}\geq 0 \end{array}$ (see Appendix II), where *k* denotes the *k*th time point, *l* and *m* denote the location (*l*, *m*) on the discrete plane, and *h* is the distance between two locations along two axes, i.e. A-Paxis (*h_x_*) and D-V axis (*h_y_*). The parameters are defined as follows: *d_i_*_,_*_l_*_,_*_m_* = *k_i_*(*x_l_*, *y_m_*)· Δ*t, w_j_*_,_*_l_*_,_*_m_* = *ϖ_j_* (*x_l_*, *y_m_*)·Δ*t a_j_*_,_*_l_*_,_*_m_* = *α_j_*(*x_l_*, *y_m_*)·Δ*t*, *b_ij_*_,_*_l_*_,_*_m_* = *β_ij_*(*x_l_*, *y_m_*)·Δ*t, c_j_*_,_*_l_*_,_*_m_* = 1 – *λ_j_*(*x_l_*, *y_m_*)· Δ*t*, and *ρ_j_*_,_*_l_*_,_*_m_* = *γ_j_*(*x_l_*, *y_m_*)·Δ*t* where Δ*t* ≈ 2.568 minutes. Then, by using the discrete 3-DEST model in Eq. (2) and mRNA and protein data, the parameters *k_i_*(*x_l_*, *y_m_*), *ϖ_j_*(*x_l_*, *y_m_*), *α_i_*(*x_l_*, *y_m_*), *β_ij_*(*x_l_*, *y_m_*), *λ_j_*(*x_l_*, *y_m_*) and *γ_j_*(*x_l_*, *y_m_*) in Eq. (2) can be estimated by the system identification method in a spatial region one by one, which will be described in the sequel. Therefore, before the system identification of discrete 3-DEST model in Eq. (2), we need to define the 2-D spatial regions of *Drosophila* embryo in the following section.

### Specification of 2-D spatial regions of *Drosophila* embryo

**Figure 1.**
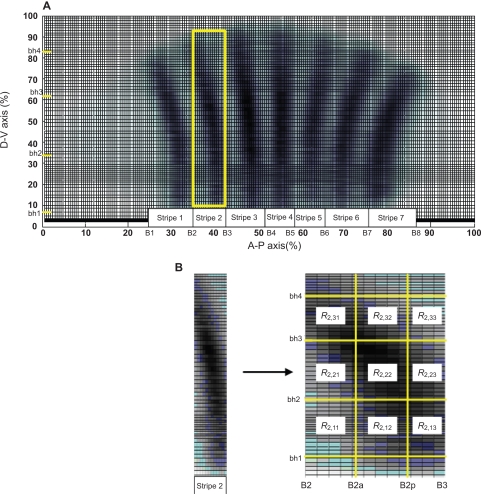
**Determination of eve stripe boundaries at c14A8. A**) Along the A-P axis and D-V axis, seven eve stripe boundaries {*B*1, *B*2, ..., *B*8} and three spatial region boundaries {*bh*1, *bh*2, *bh*3, *bh*4} are defined, respectively. **B**) The yellow square frame as shown in (*a*) is enlarged for the second eve stripe. Nine spatial regions with symbol *R*_*stripe,lk*_ are defined in each stripe.

To identify the discrete 3-DEST model in Eq. (2), we have to define (*l*, *m*) as the center of the spatial regions of the embryo by specifying the boundaries of the spatial regions. Along the A-P axis, two boundaries of the *i*th eve stripe are denoted by *Bi* and *Bi* + 1, respectively. The boundaries of seven eve stripes along the A-P axis are denoted by {*B*1, *B*2, ..., *B*8} (Fig. 1a). Each of the eve stripes along the A-P axis is separated into three parts, and the boundaries of middle part of the eve stripe *i* are denoted as *Bia* and *Bip* (Fig. 1b). Therefore, there are totally 21 spatial regions (i.e. *m* + 1, 2, ..., 21 in Eq. (2)) within seven eve stripes along the A-P axis, and the 22 boundaries of the 21 spatial regions are specified as follows:

{*B*1, *B*1*a*, *B*1*p*, *B*2, *B*2*a*, *B*2*p*, …, *B*7 *B*7*a*, *B*7*p*, *B*8} = {25%, 29%, 32.5%, 35%, 38.26%, 40.44%, 42.17%, 47%, 49%, 50%, 54.5%, 55.5%, 57.5%, 62%, 64%, 67%, 69%, 72%, 75%, 79%, 81%, 85%}.

Additionally, three spatial regions (i.e. *l* = 1, 2, 3 in Eq. (2))along the D-Vaxis are defined with their boundaries {*bh*1, *bh*2, *bh*3, *bh*4} = {8.54%, 33.67%, 61.40%, 82.25%} (Fig. 1b). For the convenience of illustration, we define a symbol, *R_stripe_*_,_*_lk_*, to be a spatial region of the location (*l*, *k*) in the *stripe*-th eve stripe. The transformation from (*l*, *m*) in the whole embryo to (*l*, *k*) in the *stripe*-th eve stripe, i.e. *R_stripe_*_,_*_lk_*, is given by *m* = *k* + 3*(*stripe*-1). For example, (*l*, *k*) = (3, 3) in the second eve stripe, i.e. the spatial region *R*_2,33_ corresponds to (*l*, *m*) = (3, 6) in the whole embryo, with *l* = 3 and *m* = 3 + 3*(2–1) = 6 (Fig. 1b). After the determination of the spatial regions, expression levels of protein and mRNA are interpolated to the determined spatial regions, which will be used for model identification of Eq. (1).

### System identification for stochastic 3-DEST gene/protein interaction networks in different spatial regions of *Drosophila* embryo

When the data points {*X_i_*(*k*, *l*, *m*), *Y_j_*(*k*, *l*, *m*)} for *i*,*j* ∈ {1, 2, ..., 14}, *k* ∈ {1,2, ..., *N*}, *l* = {1, 2, 3}, *m* = {1, ..., 21} are ready, the parameters of stochastic 3-DEST model can be estimated using Eq. (2) for gene/protein interaction networks in each spatial region of *Drosophila* embryo. For the convenience of parameter estimation, Eq. (2) with N data points can be translated into the following linear regression matrix form:

(3) $$Y_{l,m}=\Phi_{l,m}\Theta_{l,m}+E_{l,m},l=\{1,2,3\},m=\{1,2,\ldots ,21\}$$

where

$$\begin{array}{l} Y_{l,m}=\left[\begin{array}{c} \chi_{1,l,m}\\ \chi_{2,l,m}\\ \vdots \\ \chi_{14,l,m}\\ \psi_{1,l,m}\\ \psi_{2,l,m}\\ \vdots \\ \psi_{14,l,m} \end{array}\right],\;\Phi_{l,m}=\left[\begin{array}{cccccc} H_{l,m} & 0 & -Z_{l,m} & 0 & 0 & \Sigma {ϒ}_{l,m}\\ 0 & H_{l,m} & Z_{l,m} & {ϒ}_{l,m} & \Delta {ϒ}_{l,m} & 0 \end{array}\right],\;\Theta_{l,m}=\left[\begin{array}{c} D_{l,m}\\ \Omega_{l,m}\\ M_{l,m}\\ N_{l,m}\\ \Xi_{l,m}\\ O_{l,m} \end{array}\right],\\ E_{l,m}=\left[\begin{array}{c} \xi_{1,l,m}\\ \xi_{2,l,m}\\ \vdots \\ \xi_{14,l,m}\\ \varsigma_{1,l,m}\\ \varsigma_{2,l,m}\\ \vdots \\ \varsigma_{14,l,m} \end{array}\right],\;\chi_{i,l,m}=\left[\begin{array}{c} X_{i}(2,l,m)-X_{i}(1,l,m)\\ X_{i}(3,l,m)-X_{i}(2,l,m)\\ \vdots \\ X_{i}(N,l,m)-X_{i}(N-1,l,m) \end{array}\right],\;\psi_{i,l,m}=\left[\begin{array}{c} Y_{j}(2,l,m)\\ Y_{j}(3,l,m)\\ \vdots \\ Y_{j}(N-1,l,m) \end{array}\right],\\ H_{l,m}=\text{block}\;\text{diag}(1_{N-1},\;1_{N-1},\ldots 1_{N-1}),\;Z_{l,m}=\\ {}[\begin{array}{cccc} X_{1}(1,l,m) & 0 & \cdots & 0\\ X_{1}(2,l,m) & \vdots & \vdots & \vdots \\ \vdots & \vdots & \vdots & \vdots \\ X_{1}(N-1,l,m) & 0 & \vdots & \vdots \\ 0 & X_{2}(1,l,m) & \vdots & \vdots \\ \vdots & X_{2}(2,l,m) & \vdots & \vdots \\ \vdots & \vdots & \vdots & \vdots \\ \vdots & X_{2}(N-1,l,m) & 0 & \vdots \\ \vdots & 0 & \ddots & 0\\ \vdots & \vdots & 0 & X_{14}(1,l,m)\\ \vdots & \vdots & \vdots & X_{14}(2,l,m)\\ \vdots & \vdots & \vdots & \vdots \\ 0 & \cdots & 0 & X_{14}(N-1,l,m) \end{array}],\\ {ϒ}_{l,m}=\left[\begin{array}{cccc} Y_{1}(1,l,m) & 0 & \cdots & 0\\ Y_{1}(2,l,m) & \vdots & \vdots & \vdots \\ \vdots & \vdots & \vdots & \vdots \\ Y_{1}(N-1,l,m) & 0 & \vdots & \vdots \\ 0 & Y_{2}(1,l,m) & \vdots & \vdots \\ \vdots & Y_{2}(2,l,m) & \vdots & \vdots \\ \vdots & \vdots & \vdots & \vdots \\ \vdots & Y_{2}(N-1,l,m) & 0 & \vdots \\ \vdots & 0 & \ddots & 0\\ \vdots & \vdots & 0 & Y_{14}(1,l,m)\\ \vdots & \vdots & \vdots & Y_{14}(2,l,m)\\ \vdots & \vdots & \vdots & \vdots \\ 0 & \cdots & 0 & Y_{14}(N-1,l,m) \end{array}\right],\;\Delta {ϒ}_{l,m}=\left[\begin{array}{cccc} \delta {ϒ}_{1,l,m} & 0 & \cdots & 0\\ 0 & \delta {ϒ}_{2,l,m} & \ddots & \vdots \\ \vdots & \ddots & \ddots & 0\\ 0 & \cdots & 0 & \delta {ϒ}_{14,l,m} \end{array}\right],\\ \Sigma {ϒ}_{i,m}=\left[\begin{array}{cccc} \sigma {ϒ}_{1,l,m} & 0 & \cdots & 0\\ 0 & \sigma {ϒ}_{2,l,m} & \ddots & \vdots \\ \vdots & \ddots & \ddots & 0\\ 0 & \cdots & 0 & \sigma {ϒ}_{14,l,m} \end{array}\right]\\ \delta {ϒ}_{i,l,m}=[\begin{array}{c} \frac{Y_{i}(1,l-1,m)-2Y_{i}(1,l,m)+Y_{i}(1,l+1,m)}{h_{x}^{2}}+\frac{Y_{i}(1,l,m-1)-2Y_{i}(1,l,m)+Y_{i}(1,l,m+1)}{h_{y}^{2}}\\ \frac{Y_{i}(2,l-1,m)-2Y_{i}(2,l,m)+Y_{i}(2,l+1,m)}{h_{x}^{2}}+\frac{Y_{i}(2,l,m-1)-2Y_{i}(2,l,m)+Y_{i}(2,l,m+1)}{h_{y}^{2}}\\ \vdots \\ \frac{Y_{i}(N-1,l-1,m)-2Y_{i}(N-1,l,m)+Y_{i}(N-1,l+1,m)}{h_{x}^{2}}+\frac{Y_{i}(N-1,l,m-1)-2Y_{i}(N-1,l,m)+Y_{i}(N-1,l,m+1)}{h_{y}^{2}} \end{array}],\\ \sigma {ϒ}_{i,l,m}=\left[\begin{array}{cccc} Y_{1}(1,l,m) & Y_{2}(1,l,m) & \cdots & Y_{14}(1,l,m)\\ Y_{1}(2,l,m) & Y_{2}(2,l,m) & \cdots & Y_{14}(2,l,m)\\ \vdots & \vdots & \cdots & \vdots \\ Y_{1}(N-1,l,m) & Y_{2}(N-1,l,m) & \cdots & Y_{14}(N-1,l,m) \end{array}\right],\;D_{l,m}=\left[\begin{array}{c} d_{1,l,m}\\ d_{2,l,m}\\ \vdots \\ d_{14,l,m} \end{array}\right],\;\Omega_{l,m}=\left[\begin{array}{c} w_{1,l,m}\\ w_{2,l,m}\\ \vdots \\ w_{14,l,m} \end{array}\right],\\ M_{l,m}=\left[\begin{array}{c} a_{1,l,m}\\ a_{2,l,m}\\ \vdots \\ a_{14,l,m} \end{array}\right],\;N_{l,m}=\left[\begin{array}{c} c_{1,l,m}\\ c_{2,l,m}\\ \vdots \\ c_{14,l,m} \end{array}\right],\;\Xi_{l,m}=\left[\begin{array}{c} \rho_{1,l,m}\\ \rho_{2,l,m}\\ \vdots \\ \rho_{14,l,m} \end{array}\right],\\ O_{l,m}=\left[\begin{array}{c} o_{1,l,m}\\ o_{2,l,m}\\ \vdots \\ o_{14,l,m} \end{array}\right],\;o_{i,l,m}=\left[\begin{array}{c} b_{i1,l,m}\\ b_{i2,l,m}\\ \vdots \\ b_{i14,l,m} \end{array}\right],\;\xi_{i,l,m}=\left[\begin{array}{c} {ɛ}_{i}(2,l,m)\\ {ɛ}_{i}(3,l,m)\\ \vdots \\ {ɛ}_{i}(N,l,m) \end{array}\right],\;\text{and}\;\;\;\;\varsigma_{j,l,m}=\left[\begin{array}{c} \delta_{i}(2,l,m)\\ \delta_{i}(3,l,m)\\ \vdots \\ \delta_{j}(N-1,l,m) \end{array}\right] \end{array}$$

Suppose the noise components *ɛ_i_*(*k*, *l*, *m*) and δ*_j_*(*k*, *l*, *m*) are normally distributed, and the noise matrix *E_l_*_,_*_m_* has an unknown covariance matrix ∑*_l_*_,_*_m_* to be estimated. Then we use the ML method to solve the parameter estimation problem with the optimum solution Θ̂*_l_*,*_m_* and ∑̂*_l,m_*. The likelihood function of *Y_l_*_,_*_m_* is defined as follows:^41^

(4) $$\begin{array}{l} p_{l,m}(Y_{l,m}|\Theta_{l,m},\Sigma_{l,m})=\frac{1}{\sqrt{2\pi }}\text{det}{\left(\Sigma_{l,m}\right)}^{-M/2}\\ \;\;\;\;\;\;\;\;\;\;\;\;\;\;\;\;\;\;\;\;\;\;\;\;\;\;\;\;\;\;\;\;\;\;\;\;\;\;\;\;\;\;\;\;\;\text{exp}\{-\frac{1}{2}{\left[Y_{l,m}-\Phi_{l,m}\Theta_{l,m}\right]}^{T}\\ \;\;\;\;\;\;\;\;\;\;\;\;\;\;\;\;\;\;\;\;\;\;\;\;\;\;\;\;\;\;\;\;\;\;\;\;\;\;\;\;\;\;\;\;\;\times \Sigma_{l,m}^{-1}[Y_{l,m}-\Phi_{l,m}\Theta_{l,m}]\} \end{array}$$

The log-likelihood function for the given *M* data points in *Y_l_*_,_*_m_*, i.e. *M* = 2·14(*N –* 1), can be defined as^41^

(5) $$\begin{array}{l} L_{l,m}(\Theta_{l,m},\Sigma_{l,m})=\text{constant}\;-\frac{M}{2}\text{ln}[\text{det}(\Sigma_{l,m})]\\ \;\;\;\;\;\;\;\;\;\;\;\;\;\;\;\;\;\;\;\;\;\;\;\;\;\;\;\;\;\;\;-\frac{1}{2}{\left[Y_{l,m}-\Phi_{l,m}\Theta_{l,m}\right]}^{T}\\ \;\;\;\;\;\;\;\;\;\;\;\;\;\;\;\;\;\;\;\;\;\;\;\;\;\;\;\;\;\;\;\;\Sigma_{l,m}^{-1}[Y_{l,m}-\Phi_{l,m}\Theta_{l,m}] \end{array}$$

We can estimate the unknown parameters Θ*_l_*_,_*_m_* and the covariance matrices of noise ∑*_l_*_,_*_m_* by maximizing the log-likelihood function *L_l_*_,_*_m_*(Θ*_l_*_,_*_m_*, ∑*_l_*_,_*_m_*), i.e. $\frac{\partial L_{l,m}(\Theta_{l,m},\Sigma_{l,m})}{\partial \Theta_{l,m}}=0$ and $\frac{\partial L_{l,m}(\Theta_{l,m},\Sigma_{l,m})}{\partial \Sigma_{l,m}}=0$, as follows:

(6) $${\hat{\Sigma }}_{l,m}=\frac{1}{M}{\left[Y_{l,m}-\Phi_{l,m}\Theta_{l,m}\right]}^{T}[Y_{l,m}-\Phi_{l,m}\Theta_{l,m}]$$

(7) $${\hat{\Theta }}_{l,m}={\left(\Phi_{l,m}^{T}\Theta_{l,m}\right)}^{-1}\Phi_{l,m}^{T}Y_{l,m}$$

In order to satisfy the following stability constraints *d_i_*_,_*_l_*_,_*_m_* ≥ 0, *w_j_*_,_*_l_*_,_*_m_* ≥ 0, |1 – *a_j_*_,_*_l_*_,_*_m_*| ≤ 1, $\{\begin{array}{cc} |c_{j,l,m}-4\rho_{j,l,m}(h_{x}^{-2}+h_{y}^{-2})|\leq 1 & \text{if}\;\rho_{j,l,m}<0\\ \left|c_{i,l,m}\right|\leq 1 & \text{if}\;\rho_{j,l,m}\geq 0 \end{array}$ in the discrete 3-DEST model (Eq. 2), a Matlab function, *lsqlin*, is used in the estimation procedure of the parameter identification of the stochastic 3-DEST model (see Appendix III). For the stochastic 3-DEST model of gene/protein interaction network in each spatial region of embryo, the number of estimated regulatory parameters is 266. We have a total of 28 dynamic equations which will be solved simultaneously. To avoid overfitting in parameter estimation and to find a more robust solution, we should interpolate these data points by the cubic spline method. Hence, we will test the robustness of system parameters on different numbers of interpolating data points from four times to six times the number of estimated parameters in the sequel.

According to the Akaike Information Criterion (AIC) method, we will let *b_ij_*_,_*_l_*_,_*_m_* = 0 while the transcription regulation between the *j*th transcription factor and the *i*th target gene in the spatial region *R_stripe_*_,_*_lk_* is insignificant. We use the AIC to prune some insignificant regulatory parameters of TFs in Eq. (7). The AIC is defined to include both the residual variance in parameter estimation and the model complexity into one statistics for model order detection as^41^

(8) $$AIC_{l,m}(p)=\text{log}\left(\frac{1}{M}{\left(Y_{l,m}-{\hat{Y}}_{l,m}\right)}^{T}(Y_{l,m}-{\hat{Y}}_{l,m})\right)+\frac{2p}{M}$$

where *p* is the number of reserved parameters in the backward elimination method of the AIC. Regulatory parameters are pruned one by one as *p* is decreased until the smallest *AIC_l_*_,_*_m_* in the smaller *p* is larger than the *AIC_l_*_,_*_m_* value of the previous pruning step. While the minimum *AIC_l_*_,_*_m_* is achieved, the most adequate transcription regulations for each target gene could be obtained from the most adequate model order point of view.^53^

## Data and Materials

In this study, we incorporate two spatial-temporal data, protein data (http://flyex.ams.sunysb.edu/flyex/)^13^–^16^ and mRNA data (http://flyex.ams.sunysb.edu/lab/gaps.html),^4^,^6^ into the stochastic 3-DEST model to investigate how the transcription regulations and diffusion mechanisms cooperatively pattern eve stripes in the early embryogenesis of *Drosophila*. The spatial regions are first defined as shown in Figure 1 by Eve at cleavage cycle 14A and temporal class 8 (c14A8) in the embryo. Subsequently, the NN model combined with the method is trained by the available protein and mRNA data to simulate and mimic the unavailable mRNA data. The training of the NN model by the available data is achieved by minimizing the training error and maximizing the output correlations. Additionally, to avoid overfitting in system identification, we must interpolate the data points to an adequate number. However, over-interpolated data will lose the low-frequency (or long-range) behavior of the development system. Moreover, using different numbers of interpolated data in system identification may also cause significant differences in parameter estimations, especially in the AIC method. Hence, the robustness of the stochastic 3-DEST model will also be tested by different numbers of interpolated data as an assessment to choose an adequate number of interpolation in the sequel, because the robustness principle has been employed to check if a model can work in the real cell and is employed to narrow down the range of models to the few in the modeling procedure of biological networks, i.e. robustness can help theorists identify the correct dynamic model.^39^ From the ML parameter estimation method, the dynamic model in early *Drosophila* development is constructed. Then, we incorporate the AIC method into the identification process to prune the insignificant regulatory parameters and refine the model. This allows us to pick up the TFs, which are the most significant regulators for controlling the downstream genes in the early development of *Drosophila*.

The real biological systems are always robust. Therefore the model of a biological system should be robust and the robustness is a validation of dynamic models for biological systems.^39^ To test the robustness of the 3-DEST model by different number of data points, we interpolate the time-course data from 38 data points (i.e. four times the number of parameters) to 57 data points (i.e. six times the number of parameters), i.e. there are 20 test cases. However, among the 20 test cases only six test cases, which are respectively those with 38, 39, 40, 41, 42 and 44 interpolated data points, meet the model’s stability constraints *d_i_*_,_*_l_*_,_*_m_* ≥ 0, *w_j_*_,_*_l_*_,_*_m_* ≥ 0, |1 – *a_j_*_,_*_l_*_,_*_m_*| ≤ 1, $\{\begin{array}{cc} |c_{j,l,m}-4\rho_{j,l,m}(h_{x}^{-2}+h_{y}^{-2})|\leq 1 & \text{if}\;\rho_{j,l,m}<0\\ \left|c_{i,l,m}\right|\leq 1 & \text{if}\;\rho_{j,l,m}\geq 0 \end{array}$ in Eq. (2), when the AIC values of the model are minimized. Therefore, only six kinds of data interpolations to meet robustness test can be used for the parameter identification of the 3-DEST model. Here, only the robustness test of the 3-DEST system in the spatial region *R*_2,22_ is discussed for further choice of data interpolations. The robustness of the estimated basal levels and the regulatory abilities in the six test cases in the spatial region *R*_2,22_ are shown in Table 1. As can be seen, there are a few changes such as basal levels (*κ̂_i_* (*x*_2_, *y*_5_)) of *runt* at N = 39 and *hairy* at N = 41 (N represents number of interpolated data points), and there is no significant change in basal levels of protein (ϖ̂*_j_* (*x*_2_, *y*_5_)). In addition, the regulatory abilities (*β̂_eve_*_,_*_j_* (*x*_2_, *y*_5_)) of both *runt* and *slp* at N = 38 and 39 are pruned by the AIC method when compared with the others. Therefore, except a few variations in N = 38, 39 and 41 the other cases, i.e. N = 40, 42 and 44, are robust for system identification. Here, mRNA and protein data with 44 interpolation time points are chosen for parameter estimation of the stochastic 3-DEST model of the whole embryo. When spatio-temporal data are ready and the number of interpolated data points is decided (N = 44), system identification for parameter estimation in Eqs. (6)(7) and (8) can be performed.

## Results

After the parameters in Eq. (1) are estimated by ML and pruned by the AIC in Eqs. (6)–(8), the identified 3-DEST models for gene/protein interaction networks in the spatial regions of *Drosophila* embryo are given in the following.

(9) $$\begin{array}{l} \frac{\partial X_{i}(t,x_{l},y_{m})}{\partial t}={\hat{\kappa }}_{i}(x_{l},y_{m})\\ \;\;\;\;\;\;\;\;\;\;\;\;\;\;\;\;\;\;\;\;\;\;\;\;\;\;\;-{\hat{\alpha }}_{i}(x_{l},y_{m})X_{i}(t,x_{l},y_{m})\\ \;\;\;\;\;\;\;\;\;\;\;\;\;\;\;\;\;\;\;\;\;\;\;\;\;+\sum_{j=1}^{M}{\hat{\beta }}_{ij}(x_{l},y_{m})f(Y_{j}(t-\tau_{j},x_{l},y_{m}))\\ \;\;\;\;\;\;\;\;\;\;\;\;\;\;\;\;\;\;\;\;\;\;\;\;\;+\varphi_{i}(t,x_{l},y_{m})\\ \frac{\partial Y_{j}(t,x_{l},y_{m})}{\partial t}={\hat{\varpi }}_{j}(x_{l},y_{m})\\ \;\;\;\;\;\;\;\;\;\;\;\;\;\;\;\;\;\;\;\;\;\;\;\;\;\;\;\;\;+{\hat{\alpha }}_{j}(x_{l},y_{m})X_{j}(t,x_{l},y_{m})\\ \;\;\;\;\;\;\;\;\;\;\;\;\;\;\;\;\;\;\;\;\;\;\;\;\;\;\;\;\;-{\hat{\lambda }}_{j}(x_{l},y_{m})Y_{j}(t,x_{l},y_{m})\\ \;\;\;\;\;\;\;\;\;\;\;\;\;\;\;\;\;\;\;\;\;\;\;\;\;\;\;\;\;+{\hat{\gamma }}_{j}(x_{l},y_{m})\nabla^{2}Y_{j}(t,x_{l},y_{m})\\ \;\;\;\;\;\;\;\;\;\;\;\;\;\;\;\;\;\;\;\;\;\;\;\;\;\;\;\;\;+\varphi_{j}(t,x_{l},y_{m}), \end{array}$$

where *i*, *j* = 1, 2, ..., 14, *l* = 1, 2, 3, *m* = 1, 2, ..., 21. *κ̂_i_* *ϖ̂_j_*, *α̂_i_*, *β̂_ij_*, *λ̂_j_*, and *γ̂_j_* are estimated by Eq. (7) and the covariance matrices of the stochastic noises *ϕ_i_* and *ϕ_j_* can be estimated in Eq. (6).

**Figure 3.**
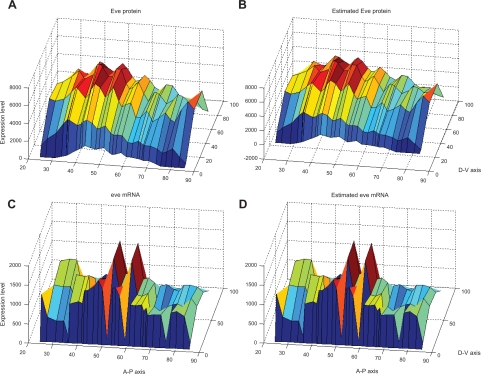
**Original data and estimated results by our proposed dynamic model.** The original eve mRNA and protein spatial data at c14A8 are shown in **A**) and **C**), respectively. After system identification, the estimated eve mRNA and protein spatial data generated by the dynamic model are shown in **B**) and **D**), respectively.

**Figure 4.**
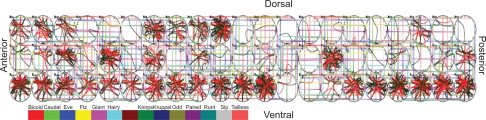
**3-DEST dynamic gene/protein interaction network for diffusion and transcriptional regulation mechanisms in different spatial regions in the whole embryo.** The notations, *R*_1,11_, *R*_1,12_, *R*_1,13_, *R*_1,21_, ..., *R*_7,31_, *R*_7,32_, *R*_7,33_, are the 63 spatial regions of the whole embryo which is specified by Figure 1(a). In each spatial region *R_stripe_*_,_*_ij_*, the colors of the outer ring in the color circle are specified by the 14 gene names, which are given by the color bar below the figure, respectively. Each color of the outer ring is specified by each gene. The solid lines that connect color circles stand for transcription regulation between genes in each spatial region based on regulatory abilities *β̂_ij_* of the identified 3-DEST dynamic model in Eq. (9). Positive and negative regulations are denoted by arrows and bars at the end of solid lines, respectively. Additionally, the colors of the inner circle, i.e. the black and white circle, inside the color circle stand for the TFs’ roles, i.e. donor or acceptor of the transcriptional regulation network, respectively. The bold color lines that connect the same genes in neighboring spatial regions with different roles stand for protein diffusions from donor (black inner circle) to acceptor (white inner circle) in neighboring spatial regions based on the diffusion coefficients *γ̂_j_* of the identified 3-DEST dynamic model in Eq. (9). The specification of the colors in bold color lines is consistent with the colors in the outer ring of the color circle, which are specified by the color bar. For example (see also Fig. 5a), Caudal in R_4,11_ with green color in outer ring and black color in inner circle found regulates ftz (yellow) and runt (navy blue) and plays as a donor, which can diffuse to the neighboring regions. A clearer figure is available online at the website, http://www.ee.nthu.edu.tw/bschen/Drosophila_Fig4.pdf.

After system identification, the simulation results of the system model obtained using the ML estimation method and the AIC method are shown in Figure 3(b) (protein) and 3(d) (mRNA) compared with the original data in Figure 3(a) (protein) and 3(c) (mRNA), respectively. The 3-DEST gene/protein interaction networks in different spatial regions are constructed in Figure 4 through the diffusion coefficients *γ̂_j_* and regulatory abilities *β̂_ij_* of the identified 3-DEST dynamic model in Eq. (9). The changes in these diffusion coefficients and regulatory abilities in eve stripes will be simultaneously investigated to see whether there are some cooperative effects on them, which may give a clue of eve stripe formation.

Previous research^48^ on cis-regulatory module detection shows that the enhancer element of the second eve stripe contains the binding sequences of Krüppel, Giant, Bicoid and Hunchback, and the second eve stripe can be activated by Bicoid, Hunchback, and repressed by Giant and Krüppel (Table 1 and Fig. 4).

In the analysis of eve stripe formation, the boundaries of eve stripe can be affected by diffusion from the neighboring regions where Eve serves as a donor to the regions where it plays the role of an acceptor. Figure 4 shows that Hunchback in *R*_2,22_, *R*_3,11_, *R*_3,33_, *R*_4,31_, *R*_6,11_, *R*_6,13_, and *R*_7,31_ and Knirps in *R*_1,12_, *R*_4,13_, *R*_5,23_ and *R*_7,13_ positively and negatively regulate *eve*, respectively, and Eve in these regions simultaneously serves as a donor which diffuses through and affects the boundaries, i.e. stripe 1–2, stripe 3–4, stripe 4–5, stripe 5–6 and stripe 7-terminal (Fig. 4). Therefore, it shows that stripe boundaries are broken in the embryo with *hunchback* and *knirps* double mutant and the phenotype is similar to the embryo with a strong *eve* mutant.^10^ In addition, *eve* in *R*_2,11_ and *R*_2,23_, which plays the role of donor and is repressed by Giant and Krüppel respectively, would locally affect the anterior and posterior of the second eve stripe, respectively (Fig. 4).^7^,^45^,^54^ Moreover, the effect of Giant and Krüppel respectively on the anterior and posterior of the second eve stripe should be diffusively reinforced by the same repressive transcription regulations in *R*_2,22_. In the boundaries of the third eve stripe, *eve* in *R*_3,31_ and *R*_4,31_, which is negatively regulated by Hunchback and Knirps respectively, would diffuse to and affect on anterior and posterior boundaries of the third eve stripe, respectively (Fig. 4).^7^,^45^,^54^ Moreover, we find that Giant and Hairy have no effect on the boundaries stripe 4–5 and stripe 5–6, respectively.

From the transcription regulations shown in Figure 4, we believe that most of them are new predicted except those discussed here because most of genetic studies in *Drosophila* are not easy to find direct transcription regulations without chromatin immunoprecipitation microarray (ChIP-chip) experiments. In this study, we provide a direction for other biologists at the wet experiments of transcription regulations especially in ChIP-chip experiments. For example, according to the robustness tests in Table 1, we show that eve in *R*_2,22_ is positively regulated by Ftz and Knirps and is negatively self-regulated. The robust regulations are the most probable suggestion in transcription regulations of eve stripe formation.

**Figure 5.**
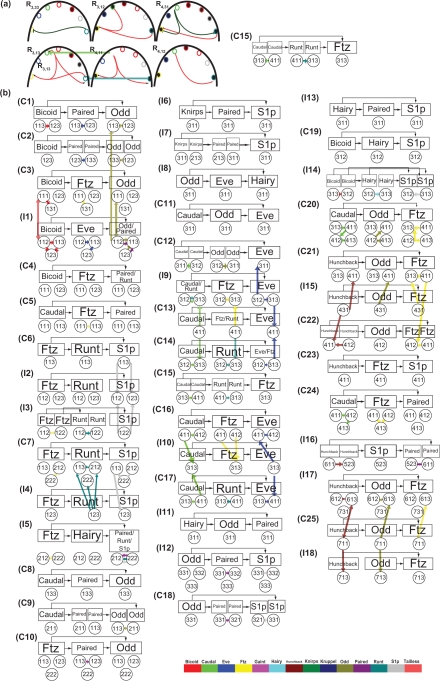
**Coherence and incoherence feedforward loops of 3-DEST dynamic gene/protein interaction network.** (**A**) According to the rule that each of the regulation relationships of FFLs must exist in at least four neighboring spatial regions, parts of gene/protein interaction network (left) in *R*_3,2_, *R*_3,3_, *R*_4,1_ and *R*_4,2_ are examples of feedforward loops, and can be redrawn as C15 (right). (**B**) From the above rule, we find the network motifs, i.e. 25 C-FFLs (C1∼C25) and 18 I-FFLs (I1∼I18), for the cooperation of transcription regulations with diffusions in early embryogenesis. The color bars denote diffusions, which are the same as those in Figure 4. A clearer figure is available online at the website, http://www.ee.nthu.edu.tw/bschen/Drosophila_Fig5.pdf.

Moreover, in the large network, there exist a huge number of interaction patterns. Only a few types of interaction patterns called network motifs, which are embedded in the network and connected to each other, allow them to carry out their functions even in the presence of additional interactions. Mangan and Alon^55^ have analyzed two feedforward network motifs, i.e. coherent feedforward loops (C-FFL) and incoherent feedforward loops (I-FFL), and found that C-FFL acted as sign-sensitive delays, and I-FFL acted as sign-sensitive accelerators.^55^ Moreover, Han et al^56^ propose that a signaling module composed of a C-FFL and an I-FFL causes an early transient response and a delayed prolonged response after a short stimulus.^56^ The early transient responses and delayed prolonged responses plausibly depend on post-translation modification of existing proteins and new protein synthesis, respectively. The combinative signaling module is suggested and found in drug therapy. Therefore, we obtain C-FFL and I-FFL from the constructed network (Fig. 5) according to the following rules. One relationship of the transcription regulations in Figure 5 serves as an edge of FFL, when the regulation relationship exists in at least four neighboring regions among its nine neighboring regions. For example (Fig. 5a), a C-FFL C15 found in Figure 5 is composed of three transcription regulations (Caudal->Ftz in *R*_3,13_, Runt->Ftz in *R*_3,13_ and Caudal->Runt in *R*_4,11_) and two diffusions (Caudal and Runt are both diffused from *R*_4,11_ to *R*_3,13_). In addition, these three regulatory relationships exist respectively in at least four neighboring regions, i.e. Caudal->Ftz in *R*_3,13_, *R*_3,12_, *R*_4,11_ and *R*_4,31_, Runt->Ftz in *R*_3,13_, *R*_3,12_, *R*_3,33_ and *R*_4,31_ and Caudal->Runt in *R*_4,11_, *R*_3,13_, *R*_4,12_ and *R*_4,31_. Therefore, C15 is one of the FFLs (Fig. 5b) found in our network. By the same procedure, not only can we find 25 C-FFLs and 18 I-FFLs (Fig. 5b) but also 13 possible combinative signaling modules among 25 C-FFLs and 18 I-FFLs, i.e. Odd in *R*_1,12_, *R*_1,11_ (C3 and I1 in Fig. 5b), *R*_1,13_ and *R*_1,23_ (C1 and I1), Slp in *R*_2,12_, *R*_2,22_ (C7 and I5), *R*_3,31_ (I12 and C18) and *R*_3,12_ (C19 and I4), Eve in *R*_3,12_, *R*_3,13_ (C14 and I9) and *R*_4,11_ (C13 and I9), and Ftz in *R*_3,13_ (C21 and I15) and *R*_6,13_ (C25 and I17). Among these modules, we find that Hunchback acts as a source of FFLs to activate Ftz as an output expressed in eve stripes 3, 4, 6 and 7. From the embryo with *hb^−^* mutants, eve stripes 2, 3, 4 and 7 are partially or completely deleted.^10^ Although Ftz in *R*_6,12_ and *R*_6,13_ is activated respectively by I-FFL and combinative signaling module with Hunchback as an input source, Ftz in *R*_6,12_ and *R*_6,13_ is respectively negatively regulated and does not regulate *eve*. Therefore, we suggest that C-FFL, I-FFL and combinative signaling module are respectively important in activating speedy responses in *R*_4,11_, *R*_4,12_ and *R*_7,11_, activating a delayed response in *R*_7,13_ with the ability of noise filtering and activating a delayed prolonged response in *R*_3,13_.

## Discussion and Conclusion

In this study, we are the first to combine mRNA dynamic equation with protein dynamic equation using spatio-temporal model to construct the gene/protein interaction network to investigate the gene/protein regulatory mechanisms of eve stripe formation in the early development of *Drosophila*. However, there are still three mechanisms of concern in *Drosophila* embryogenesis, i.e. protein-protein interactions, translation regulations and epigenetic regulations. In a recent study, protein-protein direct interactions are not found between the 14 early development-related TFs of *Drosophila* embryo,^57^ although there may exist some interactions which require a co-factor(s). For example, Bicoid has self-inhibitory property which requires a co-factor(s), and the binding site at the N-terminal region of Bicoid is evolutionarily conserved.^58^ However, the understanding of protein–protein interactions via a co-factor(s) is limited. Moreover, cooperative bindings through sigmoid function have been implicitly concerned in previous models.^38^ However, since the prior information of cooperative bindings in early embryogenesis is also limited, cooperative binding is not considered in our model. If the information of cooperative binding is most available, cooperative bindings can be considered easily as regulation candidates in the 3-DEST model, i.e. the cooperation regulation $\sum_{j,k}\beta_{ijk}f(Y_{j}(t,x,y))f(Y_{k}(t,x,y))$ could be considered in Eq. (1). In addition, there are two translation regulations of concern in early embryogenesis.^59^ The first is Bicoid which binds to maternal caudal to repress its translation,^60^,^61^ and the second is Nano which binds to the nanos response element (e.g. Pumilio) located within the 3’ untranslated region of maternal hunchback and then results in maternal hunchback, which cannot be translated.^62^–^64^ Since the understanding of translation regulations is limited so far, translation regulations are not yet included in the stochastic 3-DEST dynamic model yet. Finally, epigenetic regulations, such as DNA methylation, histone modification and RNAi, are able to play important roles in the regulation of gene expression, but they always interact to accomplish their responsibilities. Combinations of several epigenetic regulations conduct complex silencing such as chromosome inactivation and gene imprinting. For example, during *Drosophila* embryogenesis the proteins of the trithorax (trxG) and Polycomb groups (PcG) modify chromatin via interacting with chromosomal elements, Cellular Memory Modules (CMMs). A nearby gene can be continuously transcribed through mitotic cell division and meiosis by a switched activated state of CMMs during *Drosophila* embryogenesis. Thus, CMMs could affect the patterning of cells by the transcriptional control of genes involved in embryonic patterning. In conclusion, trxG and PcG confer epigenetic regulations for different binding affinities of transcription regulation, i.e. trans-effect, that result in embryonic patterning throughout *Drosophila* embryogenesis.^65^–^67^ In the 3-DEST model, the space-variant parameters of regulatory abilities *β_ij_*(*x*, *y*) and basal levels of protein generation *ϖ_j_*(*x*, *y*) have implied the affection of epigenetic regulations on transcription regulations throughout eve stripe patterning of *Drosophila* embryogenesis. An example is shown in Table 1. As seen in N = 40, 41, 42 and 44, epigenetic regulation of Hairy, which has been speculated by^68^ in the terminal system of the larvae, is probably identified that Hairy is encoded to transcriptionally regulate eve in *R*_2,22_ in eve stripe formation.

In early embryogenesis, diffusion mechanism is needed not only for maternal genes but also for gap genes and pair-rule genes to regulate their target genes in the neighboring spatial regions, which can determine the roles of TFs in each region, i.e. donor/acceptor. Without the dynamic space-time model, the dynamics of TFs’ diffusions may not be easily observed from a system point of view, especially in 2-D space. The contributions of this study include the following. (1) Construction of a stochastic 3-DEST dynamic model for gene/protein interaction network which not only contains the concentration-dependent transcriptional abilities but also includes six stochastic processes to mimic the spatio-temporal dynamic interplay among the target genes and their regulatory TFs at the early embryonic stage (i.e. the following six processes (i) protein synthesis, (ii) protein decay, (iii) mRNA decay, (iv) protein diffusion, (v) transcription regulations, and (vi) autoregulation are involved in our dynamic model). (2) Utilization of the AIC to refine the stochastic 3-DEST dynamic model for gene/protein interaction network via pruning the insignificant transcription regulations in each spatial region. (3) Findings of transcription regulations in the seven eve stripes in the stochastic 3-DEST gene/protein interaction network. (4) Validating of the identified gene/protein interaction network by literature reference with the wet experiments of gene mutations. (5) Inference of transcription regulations and diffusion mechanisms for playing a cooperative role in the creation of FFLs to build eve stripes by speedy responses, delayed responses with the ability of noise filtering and delayed and prolonged responses. For the possible experimental validation of the feedforward loops (FFLs) in 3-DEST dynamic gene/protein interaction network, biologists can follow the similar experimental design in^69^ and.^9^,^10^,^43^,^45^,^46^ For example, if the two FFLs, 
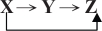
 and 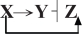 are considered, biologists can examine gene **Z**’s expression in the corresponding region found in Figure 5 of cellular blastoderm wild-type and **Y**^−^ embryos by filtered flu-orescence imaging after immunoperoxidase staining with polyclonal antibodies specific for **Z**. By comparing gene **Z**’s expression in wild-type with **Y**^−^ embryos, the suggested FFLs in Figure 5 can be validated. In the future, the proposed spatio-temporal dynamic model and construction algorithm can be extended to gene/protein network construction of different biological phenotypes, which depend on compartments, especially in early embryonic development, e.g. post-natal stem/progenitor cell regulation and differentiation, differentiation of Hematopoietic stem cells (HSCs), the segmentally modulated Hox expression patterns and patterning of the wing in *Drosophila* development.

However, one of the weaknesses in system identification is the increase in computation burden due to the use of the AIC method. Because one of our main purposes is to extract the significant transcription/translation regulations via pruning the insignificant transcription/translation regulations by using the AIC method, we use an explicit scheme with some stability constraints on the parameters to construct and then refine the gene/protein interaction network. Additionally, computation complexity will be increased, when the spatial regions are precisely specified. Moreover, a plenty of spatio-temporal data are needed in parameter estimation of the 3-DEST model. Although we know that eve stripes of the *Drosophila* embryo is probably not just built by the 14 early development-related genes, it is not a problem to estimate a more complicated dynamic regulatory network by the proposed method if much more mRNA and protein data are available in the future.

## Figures and Tables

**Figure 2.**
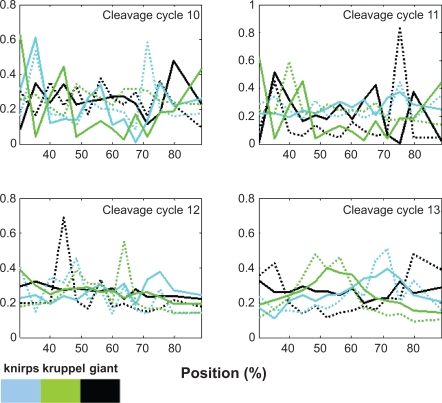
**Normalized mRNA and protein expressions.** Solid line and dashed line denote protein and mRNA expressions, respectively. The expressions of *knirps* (cyan line), *krüppel* (green line) and *giant* (black line) are plotted in time profiles.

**Table 1.** Robustness tests of parameters, κ̂*_i_* (*x_l_*, *y_m_*), *ϖ̂_j_* (*x_l_*, *y_m_*) and β̂*_eve,j_* (*x_l_*, *y_m_*) (shown in Eq. (9)), of *R*_2,22_ (i.e. *l* = 2 and *m* = *k* + 3*(*stripe*-1) = 2 + 3*(2 – 1) = 5) in the six test cases, i.e. the six test cases individually have 38, 39, 40, 41, 42 and 44 interpolated data points denoted by N = 38, N = 39, N = 40, N = 41, N = 42 and N = 44, respectively.

**Parameters**	**κ̂_*i*_(*x*_2_,*y*_5_)**						**ϖ̂_*i*_(*x*_2_,*y*_5_)**						**β̂_*eve,j*_(*x*_2_,*y*_5_)**					
		
**N**	**38**	**39**	**40**	**41**	**42**	**44**	**38**	**39**	**40**	**41**	**42**	**44**	**38**	**39**	**40**	**41**	**42**	**44**
*bicoid*	8.18	8.26	8.34	8.41	8.47	8.60	29.54	29.15	28.75	28.39	28.05	27.37	584.11	580.97	714.09	794.65	721.02	719.16
*caudal*	30.73	30.69	30.59	85.11	30.39	30.22	58.60	58.12	57.78	22.43	57.25	56.67	0	0	0	0	0	0
*eve*	0	0	0	0	0	0	0	0	0	0	0	0	−1040.69	−1063.51	−1972.63	−1881.88	−1763.69	−1590.02
*ftz*	0	0	0	0	0	0	37.59	37.69	37.79	37.88	37.96	38.08	1340.85	1343.18	3338.48	3172.99	3112.51	2913.20
*giant*	0.72	0.72	0.72	1.65	2.07	0.72	19.82	19.64	19.43	13.85	15.33	16.72	−101.86	−106.81	−445.25	−414.64	−439.09	−429.87
*hairy*	18.58	17.65	60.80	0	65.51	66.21	58.03	58.66	80.99	80.91	80.84	80.69	0	0	−1178.98	−1039.98	−1101.73	−1033.91
*hunchback*	10.32	11.45	12.41	13.13	14.03	15.65	105.69	104.42	103.70	103.26	102.40	100.84	227.98	245.25	194.56	73.79	86.04	2.15
*knirps*	3.40	3.53	3.38	3.56	3.43	3.45	5.03	4.56	5.51	4.02	4.83	4.25	163.00	164.28	638.90	546.26	566.78	505.28
*krüppel*	0.86	0.86	0.86	0.86	0.86	0.86	23.05	22.54	22.05	21.60	21.16	20.33	−1010.23	−1003.42	−2153.69	−1946.24	−1980.59	−1827.30
*odd*	0	0	0	0	0	0	0	0	0	0	0	0	0	0	0	0	0	0
*paired*	0	0	0	0	0	0	0	0	0	0	0	0	0	0	557.86	497.67	555.37	549.33
*runt*	0	17.94	0	0	0	0	0	0	0	0	0	0	0	0	589.60	467.39	522.54	465.37
*slp*	0	0	0	0	0	0	0	0	0	0	0	0	0	0	0	0	0	0
*tailless*	0	0	0	0	0	0	20.74	20.50	20.29	20.09	19.89	19.53	0	0	0	0	0	0

## Appendix I: Reconstruction of Unavailable Data by Neural-Network Learning

Because there are some deficiencies in the mRNA data, recovery of these missing data is needed when the data are used for system identification. For the deficiencies of mRNA data, a back-propagation NN training method is employed to reconstruct them. Three classes of genes, i.e. maternal genes, gap genes, and pair-rule genes, into which the 14 early development-related genes are divided, are utilized for the reconstruction of these unmeasured data in their classes of genes, individually. For each data reconstruction process, we individually train and reconstruct these data in each class of genes along the A-P axis, since the protein and mRNA data of each class of genes along the A-P axis are roughly similar (Fig. 2).^3^ Note that the downstream class of genes with missing mRNA data is reconstructed by the upstream class of genes via the back-propagation NN training method. The training methods, i.e. Broyden, Fletcher, Goldfard and Shanno (BFGS), Levenberg-Marquardt, Powell-Beale Restarte, Polak-Ribiere, Fletcher-Reeves, and Rprop, have been employed to test the performance of data reconstruction. In this study, NN combined with the BFGS method is used for training and simulating the unmeasured mRNA data,^70^ because the NN plus BFGS method has the best performance in our tests (data not shown). In order to obtain an optimal NN training results, we maximize the output correlations and minimize the training errors in the training processes. A few of the unmeasured protein data points are also reconstructed by the same learning and simulating processes. For example, if the mRNA data of gene **A** is unknown, a back-propagation NN is trained by the protein data of the upstream class of gene **A** as input and the protein data of gene **A** as output. Then the mRNA data of gene **A** can be simulated by the mRNA data of the upstream class of gene **A** through the well-trained back-propagation NN. After these missing data are simulated, the parameter estimation for the system identification of stochastic 3-DEST gene/protein interaction network model in Eq. (1) or Eq. (2) is introduced in the following section.

## Appendix II: Stability of Discrete 3-DEST Model

### Lemma 1^71^

Let $X(k,l,m)=g_{1}^{k}e^{il\Phi }e^{im\Phi }$ and $Y(k,l,m)=g_{2}^{k}e^{il\Theta }e^{im\Theta }$ where *X*(*k*, *l*, *m*) ∈ Ω*_x_* : = {*X**_i_*(*k*, *l*, *m*)} and *Y*(*k*, *l*, *m*) ∈ Ω*_y_* := {*Y**_j_*(*k*, *l*, *m*)} for *i*, *j* + 1, 2, ... 14 in Eq. (2).

An one-step finite difference scheme (with constant coefficient) is stable if and only if |*g*_1_(Φ)| ≤ 1, |*g*_2_(Θ)| ≤ 1 and *g*_1_(Φ) and *g*_2_(Θ) both are independent of *l* and *m*.

Substituting $X(k,l,m)=g_{1}^{k}e^{il\Phi }e^{im\Phi }$ and $Y(k,l,m)=g_{2}^{k}e^{il\Theta }e^{im\Theta }$ in Eq. (2), we obtain $g_{1}^{k+1}e^{il\Phi }e^{im\Phi }=(1-a_{l,m})g_{1}^{k}e^{il\Phi }e^{im\Phi }$ and

$$\begin{array}{l} g_{2}^{k+1}e^{il\Theta }e^{im\Theta }=c_{l,m}g_{2}^{k}e^{il\Theta }e^{im\Theta }+\rho_{l,m}[(g_{2}^{k}e^{i(l-1)\Theta }e^{im\Theta }\\ \;\;\;\;\;\;\;\;\;\;\;\;\;\;\;\;\;\;\;\;\;\;-2g_{2}^{k}e^{il\Theta }e^{im\Theta }+g_{2}^{k}e^{i(l+1)\Theta }e^{im\Theta })/h_{x}^{2}\\ \;\;\;\;\;\;\;\;\;\;\;\;\;\;\;\;\;\;\;\;\;\;+(g_{2}^{k}e^{il\Theta }e^{i(m-1)\Theta }-2g_{2}^{k}e^{il\Theta }e^{im\Theta }\\ \;\;\;\;\;\;\;\;\;\;\;\;\;\;\;\;\;\;\;\;\;\;+g_{2}^{k}e^{i(l+1)\Theta }e^{i(m+1)\Theta })/h_{y}^{2}]. \end{array}$$

We can obtain *g*_1_ = 1 – *a**_l_*_,_*_m_* and *g*_2_ = *c**_l_*_,_*_m_* – 4*ρ**_l_*_,_*_m_* ( $h_{x}^{-2}+h_{y}^{-2}$) sin^2^ (Θ/2).

Thus, according to Lemma 1, the scheme is stable if and only if |1 – *a**_l_*_,_*_m_*| ≤ 1 and

$$\{\begin{array}{cc} |c_{l,m}-4\rho_{l,m}(h_{x}^{-2}+h_{y}^{-2})|\leq 1 & \text{if}\;\rho_{l,m}<0\\ \left|c_{l,m}\right|\leq 1 & \text{if}\;\rho_{l,m}\geq 0 \end{array}.$$

## Appendix III: Procedure of Stability Constrained Estimation

The stability constrained estimation of parameters can be performed by a Matlab function, *lsqlin*.

A procedure for the parameter estimation is given as follows:

1. First, assume *ρ**_j_*_,_*_l_*_,_*_m_* ≥ 0 with the constraint |*c**_j_*_,_*_l_*_,_*_m_*| ≤ 1 for *j* = 1, 2, ... 14 in Eq. (2) and estimate the parameters, {*d̂**_i,l,m_*, *ŵ**_j,l,m_* *â**_i,l,m_* *ĉ**_j,l,m_* *ρ̂**_j,l,m_* *b̂**_ij,l,m_*}, under the constraints, *d**_i,l,m_* ≥ 0, *w**_i,l,m_* ≥ 0, |*c**_j_*_,_*_l_*_,_*_m_*| ≤ 1 and |1 – *a**_j_*_,_*_l_*_,_*_m_*| ≥ 1.
2. Change the assumption of *ρ**_j,l,m_* according to the estimated *ρ̂**_j,l,m_* of the previous step, and estimate the parameters under the constraints, *d**_i_*_,_*_l_*_,_*_m_* ≥ 0, *w**_j,l,m_* ≥ 0, |1 – *a**_j,l,m_*| ≤ 1 and |*c**_j,l,m_*| ≤ 1 if the diffusion coefficient estimated by the previous step for some *j* (i.e. some TFs) is non-negative, *ρ̂* ≥ 0. But otherwise the constraints of the other TFs with negative diffusion coefficients, *ρ̂**_j,l,m_* < 0, estimated by the previous step are changed to be *d**_i_*_,_*_l_*_,_*_m_* ≥ 0, *w**_j_*_,_*_l_*_,_*_m_* ≥ 0, |1 – *a**_j_*_,_*_l_*_,_*_m_*| ≤ 1 and $\left|c_{j,l,m}-4\rho_{j,l,m}(h_{x}^{-2}+h_{y}^{-2})\right|\leq 1$
3. Stop and obtain the estimated parameters if the sign of the estimated *ρ̂_j,l,m_* _,_ for each *j* is not changed anymore in the last two estimation steps, otherwise go back to step 2.

## References

[1] Driever W Nusslein-Volhard C A gradient of bicoid protein in *Drosophila* embryos Cell 1988 54 83 93 3383244 10.1016/0092-8674(88)90182-1

[2] Eldar A Dorfman R Weiss D Ashe H Shilo BZ Barkai N Robustness of the BMP morphogen gradient in *Drosophila* embryonic patterning Nature 2002 419 304 8 12239569 10.1038/nature01061

[3] Jaeger J Blagov M Kosman D Dynamical analysis of regulatory interactions in the gap gene system of *Drosophila* melanogaster Genetics 2004A 167 1721 37 15342511 10.1534/genetics.104.027334 PMC1471003

[4] Jaeger J Sharp DH Reinitz J Known maternal gradients are not sufficient for the establishment of gap domains in *Drosophila* melanogaster Mech Dev 2007 124 108 28 17196796 10.1016/j.mod.2006.11.001 PMC1992814

[5] Gilbert SF Developmental biology Sinauer Associates, Inc Publishers, Sunderland, Mass. 2006

[6] Jaeger J Surkova S Blagov M Dynamic control of positional information in the early *Drosophila* embryo Nature 2004B 430 368 71 15254541 10.1038/nature02678

[7] Wu X Vakani R Small S Two distinct mechanisms for differential positioning of gene expression borders involving the *Drosophila* gap protein giant Development 1998 125 3765 74 9729485 10.1242/dev.125.19.3765

[8] Gaul U Jackle H Role of gap genes in early *Drosophila* development Adv Genet 1990 27 239 75 1971985 10.1016/s0065-2660(08)60027-9

[9] Kraut R Levine M Spatial regulation of the gap gene giant during *Drosophila* development Development 1991 111 601 9 1893877 10.1242/dev.111.2.601

[10] Frasch M Levine M Complementary patterns of even-skipped and fushi tarazu expression involve their differential regulation by a common set of segmentation genes in Drosophila Genes Dev 1987 1 981 95 10.1101/gad.1.9.981 2892761

[11] Ingham PW Baker NE Martinez-Arias A Regulation of segment polarity genes in the *Drosophila* blastoderm by fushi tarazu and even skipped Nature 1988 331 73 5 2893285 10.1038/331073a0

[12] Jiang J Levine M Binding affinities and cooperative interactions with bHLH activators delimit threshold responses to the dorsal gradient morphogen Cell 1993 72 741 52 8453668 10.1016/0092-8674(93)90402-c

[13] Kozlov K Myasnikova E Samsonova M Reinitz J Kosman D Method for spatial registration of the expression patterns of *Drosophila* segmentation genes using wavelets Computational Technologies 2000 5 112 9

[14] Myasnikova E Samsonova A Kozlov K Samsonova M Reinitz J Registration of the expression patterns of *Drosophila* segmentation genes by two independent methods Bioinformatics 2001 17 3 12 11222257 10.1093/bioinformatics/17.1.3

[15] Myasnikova EM Kosman D Reinitz J Samsonova MG Spatio-temporal registration of the expression patterns of *Drosophila* segmentation genes Proc Int Conf Intell Syst Mol Biol 1999 195 201 10786302

[16] Surkova S Kosman D Kozlov K Characterization of the *Drosophila* segment determination morphome Dev Biol 2008 313 844 62 18067886 10.1016/j.ydbio.2007.10.037 PMC2254320

[17] Arbeitman MN Furlong EE Imam F Gene expression during the life cycle of *Drosophila* melanogaster Science 2002 297 2270 5 12351791 10.1126/science.1072152

[18] Hart CE Mjolsness E Wold BJ Connectivity in the yeast cell cycle transcription network: inferences from neural networks Plos Comput Biol 2006 2 1592 607 10.1371/journal.pcbi.0020169 PMC1761652 17194216

[19] Cheron G Draye JP Bourgeios M Libert G A dynamic neural network identification of electromyography and arm trajectory relationship during complex movements IEEE T Bio-Med Eng 1996 43 552 8 10.1109/10.488803 8849468

[20] Koike Y Kawato M Estimation of Dynamic Joint Torques and Trajectory Formation from Surface Electromyography Signals Using a Neural-Network Model Biol Cybern 1995 73 291 300 7578470 10.1007/BF00199465

[21] Linko P Zhu YH Neural Network Programming in Bioprocess Variable Estimation and State Prediction J Biotechnol 1991 21 253 69 1367695 10.1016/0168-1656(91)90046-x

[22] Liu MM Herzog W Savelberg HHCM Dynamic muscle force predictions from EMG: an artificial neural network approach J Electromyogr Kines 1999 9 391 400 10.1016/s1050-6411(99)00014-0 10597052

[23] Ressom H Reynolds R Varghese RS Increasing the efficiency of fuzzy logic-based gene expression data analysis Physiol Genomics 2003 13 107 17 12595578 10.1152/physiolgenomics.00097.2002

[24] Woolf PJ Wang YX A fuzzy logic approach to analyzing gene expression data Physiol Genomics 2000 3 9 15 11015595 10.1152/physiolgenomics.2000.3.1.9

[25] Chiang JH Chao SY Modeling human cancer-related regulatory modules by GA-RNN hybrid algorithms Bmc Bioinformatics 2007 8 91 17359522 10.1186/1471-2105-8-91 PMC1838431

[26] Maraziotis I Dragomir A Bezerianos A Recurrent neuro-fuzzy network models for reverse engineering gene regulatory interactions Lect Notes Comput Sci 2005 3695 24 34

[27] Vohradsky J Neural model of the genetic network J Biol Chem 2001 276 36168 73 11395518 10.1074/jbc.M104391200

[28] Armananzas R Inza I Larranaga P Detecting reliable gene interactions by a hierarchy of Bayesian network classifiers Comput Meth Prog Bio 2008 91 110 21 10.1016/j.cmpb.2008.02.010 18433926

[29] Djebbari A Quackenbush J Seeded Bayesian Networks: Constructing genetic networks from microarray data BMC Syst Biol 2008 2 57 18601736 10.1186/1752-0509-2-57 PMC2474592

[30] Sahoo D Dill DL Gentles AJ Tibshirani R Plevritis SK Boolean implication networks derived from large scale, whole genome microarray datasets Genome Biol 2008 9 157 10.1186/gb-2008-9-10-r157 PMC2760884 18973690

[31] Shmulevich I Dougherty ER Kim S Zhang W Probabilistic Boolean networks: a rule-based uncertainty model for gene regulatory networks Bioinformatics 2002 18 261 74 11847074 10.1093/bioinformatics/18.2.261

[32] Brown PO Botstein D Exploring the new world of the genome with DNA microarrays Nat Genet 1999 21 33 7 9915498 10.1038/4462

[33] Chang WC Li CW Chen BS Quantitative inference of dynamic regulatory pathways via microarray data BMC Bioinformatics 2005 6 44 15748298 10.1186/1471-2105-6-44 PMC555938

[34] Mestl T Plahte E Omholt SW A Mathematical Framework for Describing and Analyzing Gene Regulatory Networks Journal of Theoretical Biology 1995 176 291 300 7475117 10.1006/jtbi.1995.0199

[35] Gursky VV Reinitz J Samsonov AM How gap genes make their domains: An analytical study based on data driven approximations Chaos 2001 11 132 41 12779448 10.1063/1.1349890

[36] Perkins TJ Jaeger J Reinitz J Glass L Reverse engineering the gap gene network of *Drosophila* melanogaster PLoS Comput Biol 2006 2 e51 16710449 10.1371/journal.pcbi.0020051 PMC1463021

[37] Reinitz J Kosman D Vanario-Alonso CE Sharp DH Stripe forming architecture of the gap gene system Dev Genet 1998 23 11 27 9706690 10.1002/(SICI)1520-6408(1998)23:1<11::AID-DVG2>3.0.CO;2-9

[38] Reinitz J Sharp DH Mechanism of eve stripe formation Mech Dev 1995 49 133 58 7748785 10.1016/0925-4773(94)00310-j

[39] Alon U An introduction to systems biology: design principles of biological circuits Chapman and Hall/CRC Boca Raton, FL 2007

[40] Chen HC Lee HC Lin TY Li WH Chen BS Quantitative characterization of the transcriptional regulatory network in the yeast cell cycle Bioinformatics 2004 20 1914 27 15044243 10.1093/bioinformatics/bth178

[41] Johansson R System Modeling and Identification 1993

[42] Cadigan KM Grossniklaus U Gehring WJ Localized expression of sloppy paired protein maintains the polarity of *Drosophila* parase gments Genes Dev 1994 8 899 913 7926775 10.1101/gad.8.8.899

[43] Carroll SB Vavra SH The zygotic control of *Drosophila* pair-rule gene expression. II. Spatial repression by gap and pair-rule gene products Development 1989 107 673 83 2612385 10.1242/dev.107.3.673

[44] Schulz C Tautz D Zygotic caudal regulation by hunchback and its role in abdominal segment formation of the *Drosophila* embryo Development 1995 121 1023 8 7743918 10.1242/dev.121.4.1023

[45] Small S Blair A Levine M Regulation of two pair-rule stripes by a single enhancer in the *Drosophila* embryo Dev Biol 1996 175 314 24 8626035 10.1006/dbio.1996.0117

[46] Yu Y Pick L Non-periodic cues generate seven ftz stripes in the *Drosophila* embryo Mech Dev 1995 50 163 75 7619728 10.1016/0925-4773(94)00333-i

[47] Schroeder MD Pearce M Fak J Transcriptional control in the segmentation gene network of *Drosophila* PLoS Biol 2004 2 E271 15340490 10.1371/journal.pbio.0020271 PMC514885

[48] Rajewsky N Vergassola M Gaul U Siggia ED Computational detection of genomiccis-regulatory modules applied to body patterning in the early *Drosophila* embryo BMC Bioinformatics 2002 3 30 12398796 10.1186/1471-2105-3-30 PMC139975

[49] Brandman O Meyer T Feedback loops shape cellular signals in space and time Science 2008 322 390 5 18927383 10.1126/science.1160617 PMC2680159

[50] Klipp E Systems biology in practice: concepts, implementation and application Wiley-VCH Weinheim 2005

[51] Keener JP Sneyd J Mathematical physiology Springer New York 1998

[52] Mitchell AR Griffiths DF The finite difference method in partial differential equations Wiley, Chichester New York 1980

[53] Miller AJ Subset selection in regression Chapman and Hall/CRC Boca Raton 2002

[54] Small S Blair A Levine M Regulation of even-skipped stripe 2 in the *Drosophila* embryo Embo J 1992 11 4047 57 1327756 10.1002/j.1460-2075.1992.tb05498.x PMC556915

[55] Mangan S Alon U Structure and function of the feed-forward loop network motif Proc Natl Acad Sci U S A 2003 100 11980 5 14530388 10.1073/pnas.2133841100 PMC218699

[56] Han Z Vondriska TM Yang L Robb MacLellan W Weiss JN Qu Z Signal transduction network motifs and biological memory J Theor Biol 2007 246 755 61 17374382 10.1016/j.jtbi.2007.01.022 PMC2701969

[57] Lin CY Chen SH Cho CS Fly-DPI: database of protein interactomes for *D. melanogaster* in the approach of systems biology BMC Bioinformatics 2006 7 Suppl 5 S18 17254302 10.1186/1471-2105-7-S5-S18 PMC1764474

[58] Zhao C York A Yang F The activity of the *Drosophila* morphogenetic protein Bicoid is inhibited by a domain located outside its homeodomain Development 2002 129 1669 80 11923203 10.1242/dev.129.7.1669

[59] Niessing D Rivera-Pomar R La Rosee A A cascade of transcriptional control leading to axis determination in Drosophila J Cell Physiol 1997 173 162 7 10.1002/(SICI)1097-4652(199711)173:2<162::AID-JCP15>3.0.CO;2-I 9365516

[60] Dubnau J Struhl G RNA recognition and translational regulation by a homeodomain protein Nature 1996 379 694 9 8602214 10.1038/379694a0

[61] Rivera-Pomar R Niessing D Schmidt-Ott U Gehring WJ Jackle H RNA binding and translational suppression by bicoid Nature 1996 379 746 9 8602224 10.1038/379746a0

[62] Murata Y Wharton, RP Binding of pumilio to maternal hunchback mRNA is required for posterior patterning in *Drosophila* embryos Cell 1995 80 747 56 7889568 10.1016/0092-8674(95)90353-4

[63] St Johnston D Nusslein-Volhard C The origin of pattern and polarity in the *Drosophila* embryo Cell 1992 68 201 19 1733499 10.1016/0092-8674(92)90466-p

[64] Tautz D Regulation of the *Drosophila* segmentation gene hunchback by two maternal morphogenetic centres Nature 1988 332 281 4 2450283 10.1038/332281a0

[65] Dejardin J Cavalli G Chromatin inheritance upon Zeste-mediated Brahma recruitment at a minimal cellular memory module Embo J 2004 23 857 68 14963490 10.1038/sj.emboj.7600108 PMC381013

[66] Maurange C Paro R A cellular memory module conveys epigenetic inheritance of hedgehog expression during *Drosophila* wing imaginal disc development Genes and development 2002 16 2672 83 12381666 10.1101/gad.242702 PMC187463

[67] Rank G Prestel M Paro R Transcription through intergenic chromosomal memory elements of the *Drosophila* bithorax complex correlates with an epigenetic switch Mol Cell Biol 2002 22 8026 34 12391168 10.1128/MCB.22.22.8026-8034.2002 PMC134728

[68] Kim J Kerr JQ Min GS Molecular heterochrony in the early development of Drosophila Proc Natl Acad Sci U S A 2000 97 212 6 10.1073/pnas.97.1.212 PMC26642 10618397

[69] Mangan S Itzkovitz S Zaslaver A Alon U The incoherent feed-forward loop accelerates the response-time of the gal system of *Escherichia coli* Journal of Molecular Biology 2006 356 1073 81 16406067 10.1016/j.jmb.2005.12.003

[70] Gilbert JC Lemarechal C Some numerical experiments with variable-storage quasi-Newton algorithms Mathematical Programming 1989 45 407 35

[71] Strikwerda JC Finite difference schemes and partial differential equations Wadsworth and Brooks/Cole Advanced Books and Software Pacific Grove, Calif 1989

